# Approximate inference of gene regulatory network models from RNA-Seq time series data

**DOI:** 10.1186/s12859-018-2125-2

**Published:** 2018-04-11

**Authors:** Thomas Thorne

**Affiliations:** Department of Computer Science, University of Reading, Reading, UK

## Abstract

**Background:**

Inference of gene regulatory network structures from RNA-Seq data is challenging due to the nature of the data, as measurements take the form of counts of reads mapped to a given gene. Here we present a model for RNA-Seq time series data that applies a negative binomial distribution for the observations, and uses sparse regression with a horseshoe prior to learn a dynamic Bayesian network of interactions between genes. We use a variational inference scheme to learn approximate posterior distributions for the model parameters.

**Results:**

The methodology is benchmarked on synthetic data designed to replicate the distribution of real world RNA-Seq data. We compare our method to other sparse regression approaches and find improved performance in learning directed networks. We demonstrate an application of our method to a publicly available human neuronal stem cell differentiation RNA-Seq time series data set to infer the underlying network structure.

**Conclusions:**

Our method is able to improve performance on synthetic data by explicitly modelling the statistical distribution of the data when learning networks from RNA-Seq time series. Applying approximate inference techniques we can learn network structures quickly with only moderate computing resources.

## Background

Methods for the inference of gene regulatory networks from RNA-Seq data are currently not as mature as those developed for microarray datasets. Normalised microarray data posses the desirable property of being approximately normally distributed so that they are readily amenable to various forms of inference, and in the literature many graphical modelling schemes have been explored that exploit the normality of the data [1–9].

The data generated by RNA-Seq studies on the other hand present a more challenging inference problem, as the data are no longer approximately normally distributed, and before normalisation take the form of non-negative integers. In the detection of differential expression in RNA-Seq data, negative binomial distributions have been applied [10–13], providing a good fit to the over-dispersion typically seen in the data relative to a Poisson distribution. Following similar graphical modelling approaches as applied in the analysis of microarray data, it is natural to consider Poisson and negative binomially distributed graphical models. Unfortunately in many cases when applying graphical modelling approaches with Poisson distributed observations, only models that represent negative conditional dependencies are available, or inference is significantly complicated due to lack of conjugacy between distributions [14]. Poisson graphical models have been applied successfully in the analysis of miRNA regulatory interactions [15, 16], but we might expect to improve on these by modelling the overdispersion seen in typical RNA-Seq data sets with a negative binomial model.

One specific case of interest in the analysis of RNA-Seq data is the study of time series in a manner that takes into account the temporal relationships between data points. Previous work in the literature has developed sophisticated models for the inference of networks from microarray time series data [4, 5], but whilst methods have been developed for the analysis of differential behaviour in RNA-Seq time series [17, 18], little attention has been given to the task of learning networks from such data. Although existing nonparametric methods applicable to time series may be applied [19, 20], these were not specifically designed for application to RNA-Seq data, and also require time consuming approaches such as Markov Chain Monte Carlo schemes. There are also existing information theoretic methods, for example those of [21], but again these were designed for application to microarray data, and are not designed for time series data and learning of directed networks.

Here we present a method for the inference of networks from RNA-Seq time series data through the application of a Dynamic Bayesian Network (DBN) approach, that models the RNA-Seq count data as being negative binomially distributed conditional on the expression levels of a set of predictors. Whilst there has been work applying negative binomial regularised regression approaches in the literature [22], here we specifically consider the problem of learning networks from RNA-Seq data, and apply the horseshoe prior [23, 24], that has been shown to have advantages in robustness and adaptivity over other regularisation methods.

## Methods

### Dynamic Bayesian Networks

**Fig. 1:**
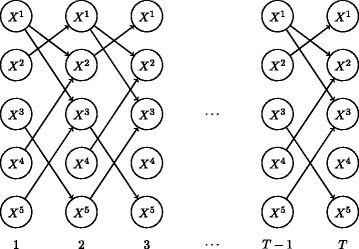
DBN of five random variables *X*^1^,…,*X*^5^ over *T* time steps. Variables are conditionally independent when conditioned on their parent variables (incoming arrows)

In a DBN framework [25], considering only edges between time points, we can model a sequence of observations using a first order Markov model, where the value of a variable at time *t* is dependent only on the values of a set of parent variables at time *t*−1. This is illustrated in Fig. 1 and can be written as

1 $$ p\left(X^{i}_{t}|X_{t-1}\right) = p\left(X^{i}_{t}|X^{\text{Pa}(i)}_{t-1}\right)  $$

where Pa(*i*) is the set of parents of variable *i* in the network. In our case we wish to model the expression level of a gene conditional on a set of parent genes that have some influence on it. To learn the set of parent variables of a given gene, it is possible to perform variable selection in a Markov Chain Monte Carlo framework, proposing to add or remove genes to the parent set in a Metropolis-Hastings sampler. Another option is to consider all possible sets of parent genes as suggested in [20]. However for even modestly sized sets of genes (e.g. 50) this can be computationally expensive, and so instead we consider applying a sparse regression approach to learn a set of parents for each gene. This approach considers the contribution of all possible parent genes in a regression framework but encourages sparsity in the coefficients so that only a small set are non-zero.

### Sparse negative binomial regression

Given data *D* consisting of *M* columns and *L* rows, with columns corresponding to genes and rows to time points, we seek to learn a parent set for each gene. To do so we can employ a regularised regression approach that enforces sparsity of the regression coefficients, and only take predictors (genes) whose coefficients are significantly larger than zero as parents. To simplify the presentation, below we consider the regression of the counts for a single gene *i*, *y*=*D*_2:*L*,*i*_, conditional on the counts of the remaining *W*=*M*−1 genes *X*=*D*_1:(*L*−1),−*i*_. The matrix *X* is supplemented with a column vector **1** to include a constant term in the regression. Where there are multiple replicates for each time point these can be adjusted appropriately.

The counts *y*_*t*_ are then modelled as following a negative binomial distribution with mean exp(*Xβ*)_*t*_ and dispersion *ω*, where *β* is a vector of regression coefficients *β*_*w*_ and a constant term *β*_*c*_. The model for a gene *i* is then

2 $$ y_{t} \sim \text{NB}\left(s_{t}\exp{(X_{t-1}\beta)_{t}},\omega\right),   $$

where we have applied the NB2 formulation of the negative binomial distribution, and *s*_*t*_ is a scaling factor for each sample to account for sequencing depth. The *s*_*t*_ can be estimated from the data by considering the sum of counts for each sample, or by the more robust approach of [11] where the median of ratios is used. We place a straightforward normal prior on *β*_*c*_ and to enforce sparsity of the *β*_*w*_ we apply a horseshoe prior [23, 24], assuming that $\beta _{w}\sim \mathcal {N}(0,\zeta ^{2}_{w})$, and placing a half-Cauchy prior on the $\zeta ^{2}_{w}$,

3 $$ \beta_{w} \sim \mathcal{N}\left(0,\zeta^{2}_{w}\right)  $$

4 $$ \zeta_{w} \sim \mathrm{C}^{+}(0,\tau).  $$

Then as in [24] we set a prior on *τ* that allows the degree of shrinkage to be learnt from the data

5 $$ \tau \sim \mathrm{C}^{+}(0,\sigma)  $$

6 $$ p\left(\sigma^{2}\right) \propto \frac{1}{\sigma^{2}}.  $$

An example of the sparsity induced in the *β*_*w*_ can be seen in figure 8 in Appendix 2. Finally we place a gamma prior on the dispersion parameter *ω*. This gives a joint probability (subsuming the model parameters into *θ*) of

7 $$ \begin{aligned} p(y,\theta|X) &= \prod_{i} p(y_{i}|X,\beta,\omega)p(\omega)\\ &\quad\, \prod_{w}p\left(\beta_{w}|\zeta^{2}_{w}\right)p\left(\zeta^{2}_{w}|\tau\right)p(\tau|\sigma)p\left(\sigma^{2}\right) \end{aligned}  $$

### Variational Inference

We now apply a variational inference [26–30] scheme to learn approximate posterior distributions over the model parameters. In a Bayesian setting variational inference aims to approximate the posterior *p*(*θ*|*x*) with a distrubtion *q*(*θ*). To do so we attempt to minimise the Kullback-Leibler (KL) divergence between the two, defined as

8 $$\begin{array}{@{}rcl@{}} \text{KL}(q(\theta)||p(\theta|x)) &=& \int q(\theta)\log\frac{q(\theta)}{p(\theta|x)} d\theta \end{array} $$

9 $$\begin{array}{@{}rcl@{}} &=& \mathbb{E}_{q}\left[\log q(\theta)\right] - \mathbb{E}_{q}\left[\log p(\theta,x)\right]\\ && + \log p(x).  \end{array} $$

As the KL divergence is bounded below by zero, it follows from 9 that

10 $$\begin{array}{@{}rcl@{}} \log p(x) &= \text{KL}(q(\theta)||p(\theta|x)) -\mathbb{E}_{q}\left[\log q(\theta)\right] \end{array} $$

11 $$\begin{array}{@{}rcl@{}}&\quad+ \mathbb{E}_{q}\left[\log p(\theta,x)\right]\\ \log p(x) &\geq \mathbb{E}_{q}\left[\log p(\theta,x)\right]-\mathbb{E}_{q}\left[\log q(\theta)\right], \end{array} $$

and so we can define a lower bound on the logarithm of the model evidence as

12 $$ \mathcal{L}(q) =\mathbb{E}_{q}\left[\log p(\theta,x)\right]-\mathbb{E}_{q}\left[\log q(\theta)\right].  $$

To make the problem of minimising the KL divergence tractable we can consider a mean field approximation where the posterior is approximated by a series of independent distributions *q*(*θ*_*i*_) on some partition of the parameters,

13 $$ p(\theta|x) \approx q(\theta) = \prod_{i} q(\theta_{i}).  $$

Under the mean field assumption it can be shown that to minimise the KL divergence between *p*(*θ*|*x*) and *q*(*θ*), or equivalently to maximise the model evidence lower bound (or ELBO) $\mathcal {L}(q)$, the optimal form for each *q*(*θ*_*i*_) is

14 $$ \log \hat{q}(\theta_{i}) = \mathbb{E}_{q_{j\neq i}}\left[\log p(\theta,x)\right] + \textrm{const.}   $$

where the expectation is over the remaining *q*(*θ*_*j*≠*i*_). In many cases this formalism is sufficient to derive a coordiante ascent algorithm to maximise the ELBO where the variational parameters of the $\hat {q}(\theta _{i})$ are updated iteratively.

Unfortunately in our model the optimal distribution $\hat {q}$ for the regression coefficients *β*_*w*_ does not have a tractable solution. However following [31] we can sidestep this problem by applying non-conjugate variational message passing [32], and we can then derive approximate posterior distributions for each of the model parameters following a straightforward parameter update scheme. The full set of variational updates are given in Appendix 1.

**Fig. 2:**
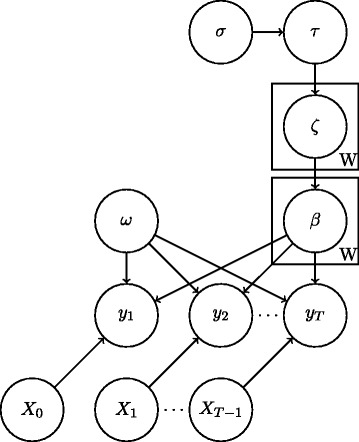
Graphical model representation of our statistical model. Applying variational message passing, the approximating distribution $\hat {q}$ of a random variable can be updated based on messages passed from connected nodes

Considering our model as a graphical model as in Fig. 2, we can decompose the terms of $\mathbb {E}_{q_{j\neq i}}\left [\log p(\theta,x)\right ]$ in Eq. 14 into those dependent on *θ*_*i*_ by considering the neighbours of *θ*_*i*_. Then we can rewrite Eq. 14 as

15 $$ {} \log\hat{q}(\theta_{i}) = \mathbb{E}_{q}\left[\log p(\theta_{i}|\theta_{\text{Pa}_{i}})\right]+\sum_{k\in\text{Ch}_{i}}\mathbb{E}_{q}\left[\log p(\theta_{k}|\theta_{\text{Pa}_{k} \neq i })\right]   $$

where Ch_*i*_ denotes the children of node *i* in the graphical model. Considering each term on the right hand side of Eq. 15 as a message from another variable in the graphical model it is possible to derive $\hat {q}$ in the conjugate exponential family as in [33]. In the non-conjugate case, the messages can be approximated as in [32], derived for the negative binomial model in [31].

## Results

### Synthetic data

We apply our method to the task of inferring directed networks from simulated gene expression time series. The time series were generated by utilising the GeneNetWeaver [34] software to first generate subnetworks representative of the structure of the *Saccharomyces cerevisiae* gene regulatory network, and then simulating the dynamics of the networks under our DBN model. Subnetworks of 25 and 50 nodes were generated and used to simulate 20 time points with 3 replicates.

Synthetic count data were generated by constructing a negative binomial DBN model as in Eq. 2 corresponding to the generated subnetworks with randomised parameters *β* sampled from a mixture of equally weighted $\mathcal {N}(0.3,0.1)$ and $\mathcal {N}(-0.3,0.1)$ distributions. The initial conditions and mean and dispersion parameters were randomly sampled from the empirically estimated means and dispersions of each gene from a publicly available RNA-seq count data set from the recount2 database [35] (accession *ERP003613*) consisting of 171 samples from 25 tissues in humans [36]. This was done so as to simulate the observed distributions of RNA-Seq counts in a real world data set.

We compare our approach against the Lasso as implemented in the lars R package [37], and the Gaussian regularised regression method in the glmnet R package [38]. For these methods the count data was first normalised, either by transforming the counts by the empirical cumulative distribution function of the data and subsequently mapping these to the quantiles of a $\mathcal {N}(0,1)$ distribution, or by applying the rlog function of the DESeq2 R package [13] to normalise the counts. We also applied the regularised Poisson regression method implemented of the glmnet R package to the count data, and the mpath R package [22] that performs penalised negative binomial regression. Finally we also applied a multinomial regularised regression from the glmnet R package to discretised data that were binned into 4 distinct levels by quantiles, to give a discrete DBN model. The degree of regularisation was in each case selected using cross validation as implemented in the respective software packages.

**Fig. 3:**
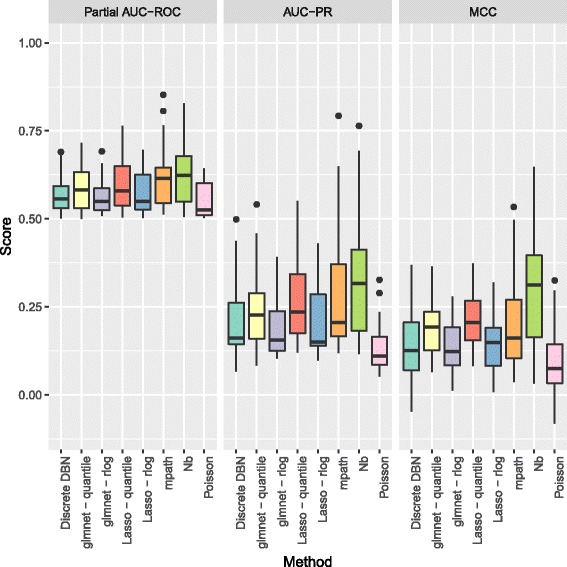
Boxplots of partial AUC-ROC, AUC-PR, and MCC for our method (Nb) and the competing methods benchmarked when learning directed networks of 25 nodes from synthetic data, for 5 subnetworks sampled from the *S. cerevisiae* gene regulatory network

**Fig. 4:**
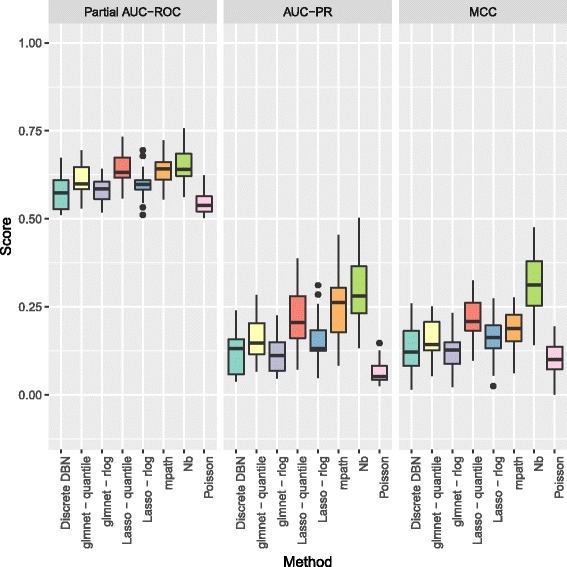
Boxplots of partial AUC-ROC, AUC-PR, and MCC for our method (Nb) and the methods benchmarked when learning directed networks of 50 nodes from synthetic data, for 5 subnetworks sampled from the *S. cerevisiae* gene regulatory network

In Figs. 3 and 4 we show the partial area under the receiver operating curve (AUC-ROC) with a cutoff of 0.95 and corrected to fit the range 0 to 1, and area under the precision recall curve (AUC-PR), as calculated by the PRROC R package [39], and Matthews Correlation Coefficient (MCC), for the various methods to be benchmarked. For the MCC, edges were predicted as those where zero was not contained in the 95% credible interval of the corresponding regression coefficients, and for the Lasso and glmnet methods, non-zero coefficients were taken as predicted edges. As the count data were generated by a stochastic model, we repeated benchmarking on each network 5 times with resampled negative binomial means and dispersions and simulated count data. Running time for our algorithm was under 10 minutes for the 50 node networks considered.

For networks of 25 nodes in Fig. 3, our method shows an improved performance over the competing methods in terms of the AUC-PR, and also in terms of the MCC. Although the distinction between the approaches is less marked for the AUC-ROC, this is to be expected as the simulated biological network structures have far fewer (< 10*%*) true positives than true negatives, a situation in which the AUC-ROC does not distinguish performance as well as AUC-PR [39, 40].

As can be seen in Fig. 4 performance for larger networks of 50 nodes is also improved over competing methods in terms of AUC-PR and MCC. For the competing methods, quantile normalisation for the Lasso and glmnet appear to outperform normalisation using the rlog function of DESeq2. As the only other method applying a negative binomial distribution, mpath is the closest method to our approach, but it appears that the application of the horseshoe shrinkage prior delivers improved performance. It is clear that, as might be expected, taking into account the distributional properties observed in RNA-Seq data improves on the performance of methods based on assumptions that do not hold for RNA-Seq count data.

## Neural progenitor cell differentiation

To illustrate an application of our model to a real world RNA-Seq data set, we consider a publicly available RNA-Seq time course data set available from the recount2 database [35], accession *SRP041159*. The data consist of RNA-Seq counts from neuronal stem cells for 3 replicates over 7 time points after the induction of neuronal differentiation [41]. To select a subset of genes to analyse we performed a differential expression test between time points using the DESeq2 R package [13], and selected the 25 genes with the largest median fold-change between time points that were also differentially expressed between all time points.

**Fig. 5:**
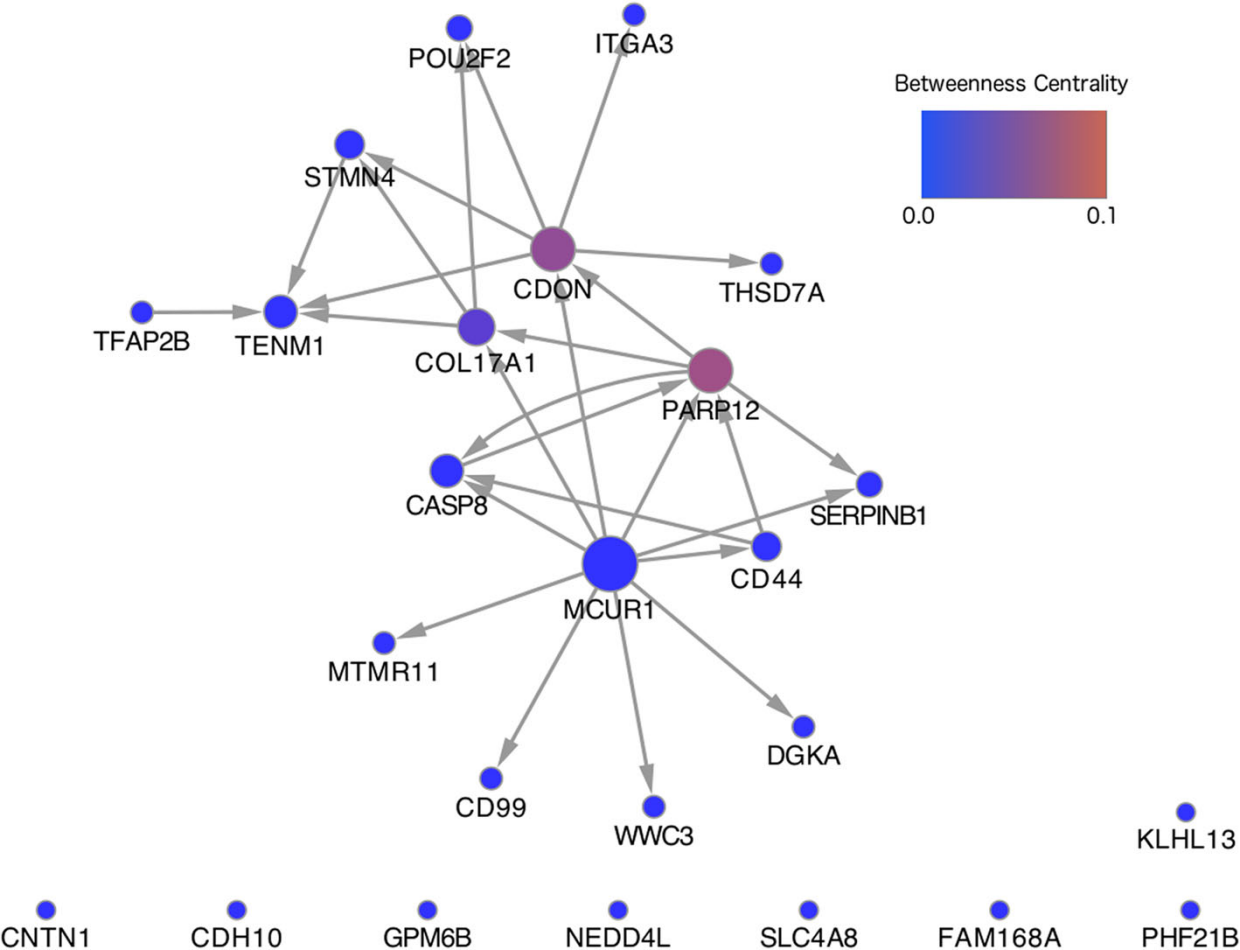
DBN inferred from the human neuronal differentiation time series data set. Edges were selected using a posterior probability cut-off of 0.95

Applying our method and selecting edges with a posterior probability > 0.95 produced the network shown in Fig. 5, where it can be seen that there are four genes (MCUR1, PARP12, COL17A1, CDON) acting as hubs, suggesting these genes may be important in neuronal differentiation. Within the network MCUR1 appears to influence the transcription of a large number of genes with many outgoing edges, whilst PARTP12, COL17A1 and CDON have both incoming and outgoing edges. This may suggest a more fundamental role of MCUR1 in controlling neuronal differentiation.

For each node we also calculate the betweenness centrality, which is the fraction of the total number of shortest paths between nodes in the network that pass through a given node. This gives a measure of the importance of a node in the network, as nodes with a larger betweeness centrality will disrupt more paths within the network if deleted, and act as bottlenecks that connect modules within the network. Looking at the betweenness centrality of each node it appears that PARP12 and CDON, and to a lesser extent COL17A1, are important carriers of information within the network. Of these genes playing a central role in the network, CDON has been shown to be promote neuronal differentiation through the activation of p38MAPK pathway [42, 43] and inhibition of Wnt signalling [44], whilst MCUR1 is known to bind to MCU [45], that in turn has been shown to influence neuronal differentiation [46].

## Discussion and conclusions

We have developed a fast and robust methodology for the inference of gene regulatory networks from RNA-Seq data that specifically models the observed count data as being negative binomially distributed. Our approach outperforms other sparse regression based methods in learning directed networks from time series data.

Another approach to network inference from RNA-Seq data could be to further develop mutual information based methodologies with this specific problem in mind. Mutual information based methods have the benefit of being independent of any specific model of the distribution of the data, and so could help sidestep issues in parametric modelling of RNA-Seq data. However this comes at the cost of abandoning the simplifying assumptions that are made by applying a parametric model that provides a reasonable fit to the data, and presents challenges of its own in the reliable estimation of the mutual information between random variables.

## Appendix 1: Variational inference

From the results in [31] the model can be written as a Poisson-Gamma mixture, so that

16 $$\begin{array}{@{}rcl@{}} p(y_{t}|\lambda_{t}) &\sim& \text{Pois}(\lambda_{t}) \end{array} $$

17 $$\begin{array}{@{}rcl@{}} p(\lambda_{t}|x_{t},\beta,\omega) &\sim& \text{Gamma}\left(\omega, \omega\exp\left[-X\beta\right]\right) \end{array} $$

and the horseshoe prior on *β* represented using a mixture of inverse gamma distributions,

18 $$\begin{array}{@{}rcl@{}} p\left(\beta_{w}|\zeta^{2}_{w}\right) &\sim& \mathcal{N}\left(0,\zeta^{2}_{w}\right) \end{array} $$

19 $$\begin{array}{@{}rcl@{}} p\left(\zeta^{2}_{w}|a_{w}\right) &\sim& \text{InvGamma}\left(\frac{1}{2},\frac{1}{a_{w}}\right) \end{array} $$

20 $$\begin{array}{@{}rcl@{}} p\left(a_{w}|\tau^{2}\right) &\sim& \text{InvGamma}\left(\frac{1}{2},\frac{1}{\tau^{2}}\right) \end{array} $$

21 $$\begin{array}{@{}rcl@{}} p\left(\tau^{2}|b\right) &\sim& \text{InvGamma}\left(\frac{1}{2},\frac{1}{b}\right) \end{array} $$

22 $$\begin{array}{@{}rcl@{}} p\left(b|\sigma^{2}\right) &\sim& \text{InvGamma}\left(\frac{1}{2},\frac{1}{\sigma^{2}}\right). \end{array} $$

### Mean field approximation

The mean field approximation of the posterior is then

23 $$ {{} \begin{aligned} &\prod_{i} p(y_i|\lambda_i)p(\lambda_i|X_{i},\beta,\omega)p(\omega)\prod_{w} p\left(\beta_w|\zeta^{2}_{w}\right)p\left(\zeta^{2}_{w}\right)p\left(\zeta^{2}_w|\tau\right)\\&\qquad\qquad\qquad\qquad\qquad\qquad\qquad p\left(\tau|\sigma^{2}\right)p\left(\sigma^{2}\right)\\ &\approx\prod_{i} \left[q(\lambda_i)\right] q(\beta) q(\omega) \prod_{w} \left[q(\zeta^{2}_w)q(a_w)\right]q\left(\tau^{2}\right)q(b). \end{aligned}}  $$

The variational updates for *λ*_*t*_ can be derived as

24 $$ {{}\begin{aligned} \log \hat{q}(\lambda_{t})&=\mathbb{E}_{q} \left[ \log p(y_{t}|\lambda_{t}) p(\lambda_{t}|X_{t},\beta,\omega)\right] + \text{const.}\\ &= \mathbb{E}_{q} \left[\!\log \frac{\lambda_{t}^{y_{t}}e^{-\lambda_{t}}}{y_{t}!}\frac{(\omega\exp(-X_{t}\beta))^{\omega}\lambda_{t}^{\omega-1} e^{-\lambda_{t} \omega\exp(-X_{t}\beta)}}{\Gamma(\omega)}\!\right] + \text{const.}\\ &= \mathbb{E}_{q} \left[y_{t}\log\lambda_{t} \,-\,\lambda_{t} \,+\, (\omega\,-\,1)\log\lambda_{t} -\lambda_{t}\omega\exp(\,-\,X_{t}\beta)\right] \,+\, \text{const.}\\ \end{aligned}}  $$

25 $${} {\begin{aligned} \hat{q}(\lambda_{t})\sim \text{Gamma}\left(y_{t}+\mathbb{E}_{q}\left[\omega\right],1+\mathbb{E}_{q}\left[\omega\right]\mathbb{E}_{q}\left[\exp(-X_{t}\beta)\right]\right) \end{aligned}}  $$

and due to the properties of the log-normal distribution

26 $${} \mathbb{E}_{q}\left[\exp(-X_{t}\beta)\right]=\exp\left(-X_{t}\mathbb{E}\left[\beta\right]+\frac{1}{2}X_{t}\Sigma X_{t}^{T}\right),  $$

where *Σ* is the covariance matrix of *β* under $\hat {q}$.

As derived in [31], applying non-conjugate variational message passing, $\hat {q}(\beta)\sim \mathcal {N}(\mu,\Sigma)$ and the variational update for *β* is

27 $$\begin{array}{@{}rcl@{}} w&=&\exp\Big(-X\mu+\frac{1}{2}\text{diagonal}~\left(X\Sigma X^{T}\right)\Big) \end{array} $$

28 $$\begin{array}{@{}rcl@{}} \Sigma&=& \left[\omega X^{T} \text{diag}(\mathbb{E}\left[\lambda\right]\cdot w) X + M\right]^{-1} \end{array} $$

29 $$\begin{array}{@{}rcl@{}} M&=&\text{diag}\left(\mathbb{E}\left[\frac{1}{\sigma^{2}_{w}}\right]\right) \end{array} $$

30 $$\begin{array}{@{}rcl@{}} \mu&=&\mu + \Sigma\left[ \omega X^{T}(\mathbb{E}\left[\lambda\right]\cdot w -1) -M\mu\right], \end{array} $$

and for the dispersion *ω* we apply numerical integration as described in [31].

Then for the horseshoe prior on *β*, the variational updates are

31 $$\begin{array}{@{}rcl@{}} {} \log \hat{q}\left(\zeta^{2}_{w}\right)&=&\mathbb{E}_{q} \left[\log p\left(\beta_{w}|\zeta^{2}_{w}\right) p\left(\zeta^{2}_{w}\right)\right] + \text{const.}\\ &=&\mathbb{E}_{q} \left[-\frac{1}{2}\log \zeta_{w}^{2}-\frac{\beta_{w}^{2}}{2\zeta^{2}_{w}} + (-\alpha-1)\log \zeta^{2}_{w}-\frac{\gamma}{\zeta^{2}_{w}} \right] \\&&+~ \text{const.} \end{array} $$

32 $$\begin{array}{@{}rcl@{}} \hat{q}\left(\zeta^{2}_{w}\right) &\sim& \text{InvGamma}\left(1,\frac{1}{2}\mathbb{E}\left[\beta_{w}^{2}\right]+\mathbb{E}_{q}\left[a_{w}\right]\right) \end{array} $$

33 $$\begin{array}{@{}rcl@{}} \log\hat{q}(a_{w}) &=& \mathbb{E}_{q}\left[\log{p\left(\zeta^{2}_{w}|a_{w}\right)p\left(a_{w}|\tau^{2}\right)}\right] + \text{const.}\\ &=& \mathbb{E}_{q}\left[-\frac{1}{a_{w}\zeta^{2}_{w}}-\frac{1}{2}\log{a_{w}}-\frac{3}{2}\log{a_{w}}-\frac{1}{\tau^{2} a_{w}} \right] \\&&+ \text{const.} \end{array} $$

34 $$\begin{array}{@{}rcl@{}} \hat{q}(a_{w}) &\sim& \text{InvGamma}\left(1,\mathbb{E}_{q}\left[\frac{1}{\zeta^{2}_{w}}\right]+\mathbb{E}_{q}\left[\frac{1}{\tau^{2}}\right]\right) \end{array} $$

35 $$\begin{array}{@{}rcl@{}} {} \log\hat{q}\left(\tau^{2}\right) &=& \mathbb{E}_{q}\left[\sum_{w} \log{p\left(a_{w}|\tau^{2}\right)} + \log{p\left(\tau^{2}|b\right)} \right] + \text{const.}\\ &=& \mathbb{E}_{q}\left[-\sum_{w}\left(\frac{1}{2}\log{\tau^{2}}+\frac{1}{a_{w}\tau^{2}}\right) - \frac{3}{2}\log{\tau^{2}}-\frac{1}{b\tau^{2}} \right] \\&&+~ \text{const.} \end{array} $$

36 $$\begin{array}{@{}rcl@{}} \hat{q}\left(\tau^{2}\right) &\sim& \text{InvGamma}\left(\frac{1}{2}+\frac{W}{2},\mathbb{E}_{q}\left[\frac{1}{b}\right]+\sum_{w}\mathbb{E}_{q}\left[\frac{1}{a_{w}}\right]\right)\\ \end{array} $$

37 $$\begin{array}{@{}rcl@{}} {} \log\hat{q}(b) &=& \mathbb{E}_{q}\left[\log{p\left(\tau^{2}|b\right)p\left(b|\sigma^{2}\right)}\right] + \text{const.}\\ &=& \mathbb{E}_{q}\left[-\frac{1}{2}\log{b}-\frac{1}{\tau^{2} b}-\frac{3}{2}\log{b}-\frac{1}{\sigma^{2} b} \right]\\&& +~ \text{const.} \end{array} $$

38 $$\begin{array}{@{}rcl@{}} \hat{q}(b) &\sim& \text{InvGamma}\left(1,\mathbb{E}_{q}\left[\frac{1}{\tau^{2}}\right]+\mathbb{E}_{q}\left[\frac{1}{\sigma^{2}}\right]\right) \end{array} $$

39 $$\begin{array}{@{}rcl@{}} \log\hat{q}\left(\sigma^{2}\right) &=& \mathbb{E}_{q}\left[\log{p\left(b|\sigma^{2}\right)p\left(\sigma^{2}\right)}\right] + \text{const.}\\ &=& \mathbb{E}_{q}\left[-\frac{1}{2}\log{\sigma^{2}}-\frac{1}{b\sigma^{2}}-\log{\sigma^{2}} \right] \\&&+~ \text{const.} \end{array} $$

40 $$\begin{array}{@{}rcl@{}} \hat{q}\left(\sigma^{2}\right) &\sim& \text{InvGamma}\left(\frac{1}{2},\mathbb{E}_{q}\left[\frac{1}{b}\right]\right). \end{array} $$

## Appendix 2: Supplemental Figures

**Fig. 6:**
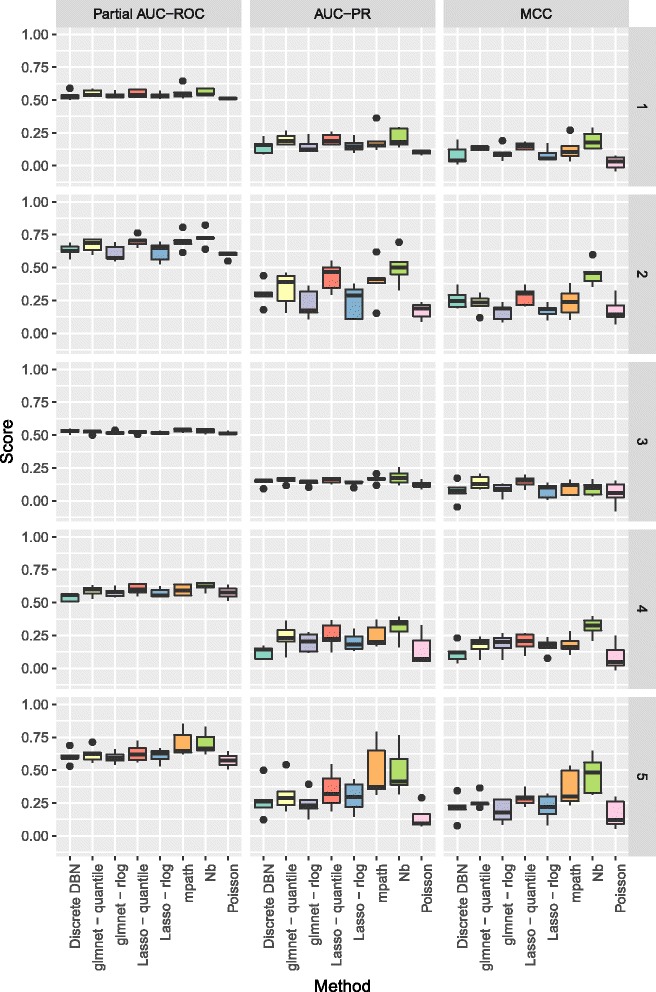
Metrics calculated for networks of 25 nodes separated by individual network structure for the 5 different networks considered. Each bar plot corresponds to 5 simulated data sets from a single network structure

**Fig. 7:**
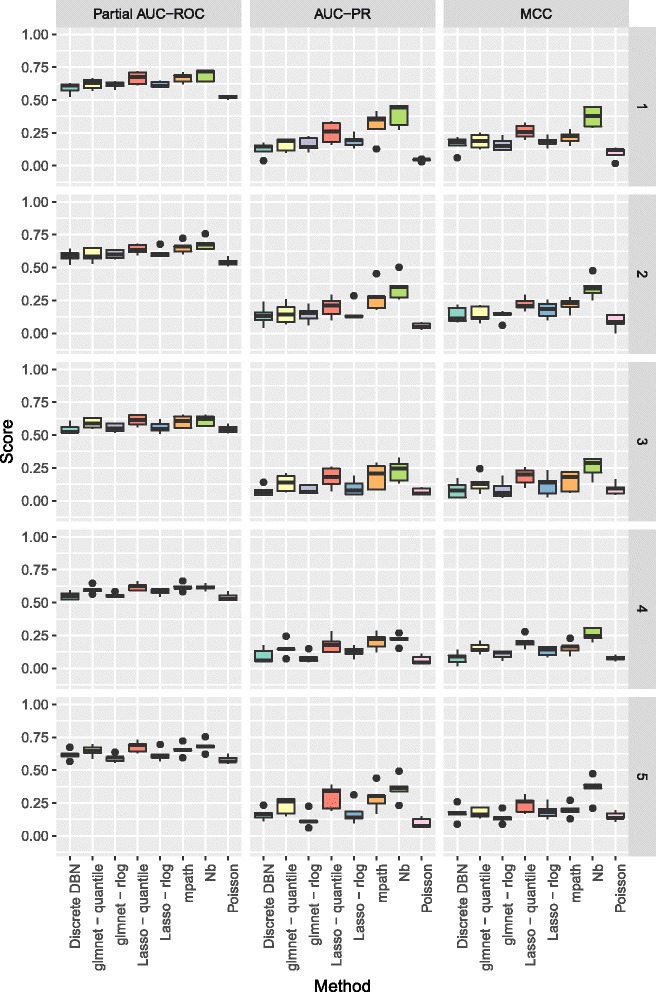
Metrics calculated for networks of 50 nodes separated by individual network structure for the 5 different networks considered. Each bar plot corresponds to 5 simulated data sets from a single network structure

**Fig. 8:**
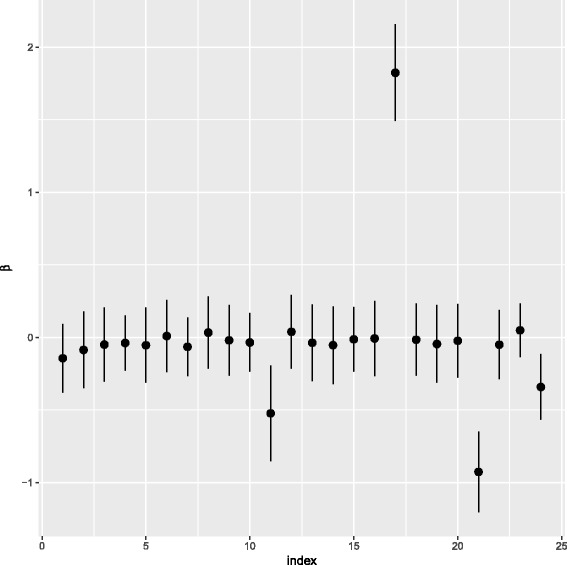
Posterior means and standard deviations for the regression coefficients *β* for a single node when applied to the NPC data considered in “Neural progenitor cell differentiation” section
